# When Women Deliver with No One Present in Nigeria: Who, What, Where and So What?

**DOI:** 10.1371/journal.pone.0069569

**Published:** 2013-07-25

**Authors:** Bolaji M. Fapohunda, Nosakhare G. Orobaton

**Affiliations:** 1 International Division, MEASURE Evaluation, John Snow, Incorporated, Rosslyn, Virginia, United States of America; 2 International Division, John Snow, Incorporated, Rosslyn, Virginia, United States of America; 3 Targeted States High Impact Project (TSHIP), Bauchi, Bauchi State, Nigeria; London School of Economics, United Kingdom

## Abstract

With the current maternal mortality ratio (MMR) of 630/100,000 live births, Nigeria ranks among the nations with the highest mortality rates in the world. The use of skilled assistants during delivery has been identified a key predictor in the reduction of mortality rates in the world over. Not only are Nigerian women predominantly using unskilled attendants, one in five births are delivered with No One Present (NOP). We assessed who, what, where and the so what of this practice using 2008 Nigeria DHS (NDHS) data. The study revealed that the prevalence of NOP is highest in the northern part of Nigeria with 94% of all observed cases. Socio-demographic factors, including, women’s age at birth, birth order, being Muslim, and region of residence, were positively associated with NOP deliveries. Mother’s education, higher wealth quintiles, urban residence, decision-making autonomy, and a supportive environment for women’s social and economic security were inversely associated with NOP deliveries. Women’s autonomy and social standing were critical to choosing to deliver with skilled attendance, which were further amplified by economic prosperity. Women’s’ economic wellbeing is entwined with their feelings of independence and freedom. Programs that seek to improve the autonomy of women and their strategic participation in sound health seeking decisions will, most likely, yield better results with improvements in women’s education, income, jobs, and property ownership. As a short term measure, the use of conditional cash transfer, proven to work in several countries, including 18 in sub-Saharan Africa, is recommended. Its use has the potential to reduce household budget constraint by lowering cost-related barriers associated with women’s ability to demand and use life-saving services. Given the preponderance of NOP in the Northern region, the study suggests that interventions to eradicate NOP deliveries must initially focus this region as priority.

## Introduction

With current maternal mortality ratio (MMR) of 630 per 100,000 live births, Nigeria ranks among the countries with the highest maternal mortality rates in the world [1]. Although maternal mortality has continued to decline since the 90 s, albeit at a slower than expected annual rate of 2·6%, the current MMR is still 40 times higher than published rates in developed countries, and is twice as high as the rates in developing countries (Table 1). World Health Organization (WHO) noted that Nigeria alone contributes 14% of all maternal deaths in the world today. Roughly one in seven of women dying of maternal related causes globally live in Nigeria. At this rate, by the end of 2013, roughly 40,000 women of reproductive age in Nigeria would have died of maternal related deaths [1]. Expressed in real life time risk, unless reversed, 1 in 29 women who are 15-years old today will die eventually from a maternal related cause in Nigeria. In contrast, the risk of dying in developing countries is 1 in 150 and 1 in 4,700 in industrialized countries [1]. The persistently high rates of maternal mortality in Nigeria are even more stark when the rates are disaggregated by political zone [2], [3]. Estimates from several studies were collated and are presented in Table 2, to illustrate variations in the picture of mortality patterns in the country [4], [5], [6], [7], [8], [9], [10]. For example, the rate is 1049 per 100,000 live births in Zamfara State [8] and 1500/100,000 live births in Sokoto State [7]. Some locality estimates based on hospital records showed a rate as high as 2,397 per 100,000 live births for the South East, Nigeria, although hospital-based rates are typically higher than population-based rates [4].

**Table 1:** Comparison of 1990, 1995, 2000, 2005 and 2010 estimates of maternal mortality ratio (MMR, maternal deaths per 100,000 live births) in Nigeria.

Year	MMR		
	Nigeria	Sub-Saharan Africa	Developing Countries
**1990**	1100	850	440
**1995**	1000	820	400
**2000**	970	740	350
**2005**	820	630	290
**2010**	630	500	240
**% Change, 1990–2010**	−41%	−41%	−47%
**Annual % Change, 1990–2010**	−2·6%	−2·6%	−3·1
**Life time risk of maternal death** *	1 in 29 women	1 in 39 women	1 in 150 women
**Progress towards improving maternal health**	**Making Progress**	Na	Na

* Adult life time risk of maternal death: the probability that a 15-year-old woman will die eventually from a maternal cause.

Source: WHO, UNICEF, UNFPA and the World Bank: 2012. Trends in Maternal Mortality: 1990–2010. Geneva: WHO (http://www.unfpa.org/webdav/site/global/shared/documents/publications/2012/Trends_in_maternal_mortality_A4-1.pdf, accessed on 27 March 2013).

**Table 2:** Illustrative locality maternal mortality estimates, based on hospital records and vital registration, Nigeria, 1987–2010.

Place	MMR	Year	Source of data
Ilorin, North Central, Nigeria	450*	1987	Adetoro OO. Int’l J of Obst 1987 [4] Hospital records on number of maternal deaths, maternal age, parity and cause of death were obtained from case notes over 12-year period,1972–1983
UNTH, Enugu, South East, Nigeria	2,397	2007	Ozumba & Iwogu-Ikojo, 2007 [5] Hospital HMIS @ the hospital; reviewedrecords of women admitted to the University of Nigeria Teaching Hospital (UNTH), Enugu, Nig. between Jan 2003-Dec 2005.
St. Vincent Hospital, South East, Nigeria	2,366	2010	Umeora & Egwuatu 2010 [6] Case study of a hospital, MMR was calculated based on data from referrals.
Maternal mortality estimates from vital registration of deaths			
Sokoto, North West, Nigeria	1500	2009	UN Fund for Population Activities (UNFPA) 2013 [7]UNFPA Technical Report, http://nigeria.unfpa.org/sokoto.html, accessed on May 27, 2013.
Zamfara, Northwest, Nigeria	1049	2012	Doctor & Olatunji, et.al. 2012 [8] Demographic Surveillance System, Columbia University, USA, involving 306 villages and 19,193 households

Globally and locally, studies have identified delivery assistance as a vitally important factor associated with positive improvements in maternal mortality rates [9], [10], [11], [12], [13]. Based on the analysis of MMR in 181 countries, Hogan and Foreman concluded that between 1990 and 2008, the increase in the proportion of women who gave birth with a skilled attendant was a main explanatory factor in the decline in global MMR. Countries such as India with large declines in MMR over the same period also experienced a concomitant increase in the proportion of deliveries made by skilled attendants. During the same period, of the 25 countries studied by Limwattananon et. al., sub-Saharan Africa recorded the smallest magnitude of decline in MMR [14].

The limited use of skilled birth attendance (SBA) has been documented in Nigeria. Prata and Ejembi found that of the 1,875 women enrolled in a community-based program to improve delivery outcomes in Zaria, Nigeria, 95% of who delivered at home, only 7% was attended by a skilled attendant [15]. Doctor and Findley *et al*., in a survey of 6,882 married women in northern Nigeria, found that only 26% of the women surveyed had received any antenatal care, 13% delivered in a facility attended by skilled birth attendant, and 86% gave birth at home under unskilled care [2]. In a cross-sectional survey of health facilities in Shagamu Local Government Area in SW Nigeria, Adelaja found that 67% of the 300 women studied delivered at home, and the majority utilized unskilled attendants. In the study, 86% of all home deliveries used unhygienic surfaces, and most utilized unclean kitchen equipment (such as knives, scissors) for cord cutting, with higher risk of infection for both mother and child [15].

Delivery outcomes are modulated by the type of delivery attendance utilized. Lawoyin and Onadeko et al. [8] contrasted the likelihood of neonates to die with skilled delivery assistance and other scenarios: neonates were five times more likely to die if they had no attendance at birth, three times more likely to die if their births were attended by Traditional Birth Attendants (TBA), and four times more likely to die if they were delivered outside the health facility. A matched case control study of 56 infants with permanent congenital and early hearing loss (PCEHL) and 280 normal hearing controls also found that infants delivered with unskilled attendance were four times more likely to develop PCEHL than their peers who were delivered with skilled attendance [16]. These findings are consistent with the results of a 10-year autopsy review in a referral hospital in Cross Rivers State, which showed that 43% of the maternal deaths occurred at the TBA centers and most were associated with preventable causes [17].

We searched the literature for reasons why many women in Nigeria continue to use any of these unsafe delivery approaches. The review identified supply and demand side factors as pivotal in the utilization of delivery attendance. In the case of unskilled attendance, the supply side factors include generalized health systems weakness, indexed by lack of infrastructure (e.g. poor or no supply of electricity) and equipment (e.g. ambulances); perennial stock out of obstetric care commodities; sub-standard emergency obstetric care (EmOC) and poor compliance with standard of practices; inadequate health worker size, mix, capacity, motivation; weak referral linkages and feedback mechanisms [17], [18]–[20]. Nyango and Mutihir et al. [21] reported that most (94%) of the health facilities (HF) they studied in Nasarawa State did not provide standard EmOC services to women. In another review of delivery practices at secondary health facilities in Nigeria, Osungbade et al. found that basic EmOC services (vaginal exam, fetal heart monitoring, and blood pressure measurement) were given to less than two-thirds of the 309 subjects, and less than adequate quality persisted well into the labor stage of delivery [22]. A study of 152 health workers (HW) (doctors, nurses, midwives, and Community Health Extension Workers (CHEWS) from 22 health facilities in five cities of two States in Nigeria found that 91% of the HW had poor knowledge of EmOC concepts, and 60% did not counsel clients on complications readiness. Only two-thirds of them adhered to the recommended EmOC standard of practice [23]. Given these facility-level deficiencies, women reportedly discerned no comparative advantage in delivery at PHCs over deliveries by TBA at home. This may explain why many women still opt for TBA delivery despite the established life-threatening limitations of TBA, notably their weak judgment [24]–[26], [27]–[30]. Other access related issues include high user-fees, limited health facilities within reasonable commuting distance, and poor HW attitudes, including their lack of respect in care-giving. Furthermore, the fear of stigma and loss of privacy were additional reasons many pregnant women living with HIV/AIDS (PLWHAs) do not use skilled birth attendance in Nigeria [31], [32], [33], [34].

Demand side factors in maternal health care include cost of services, prohibitive local customs; husbands/significant others that are not supportive of use of formal services; unwillingness by some women to see a male health care provider; limited knowledge of available services by others [26]; poor socioeconomic status, and the persistence of gender norms that are adversarial to women’s health [27]–[29]. In a recent study, Singh et al. examined the effects of socioeconomic and gender factors on the utilization of facility delivery in Nigeria. The study found that factors such as women’s age, education, higher wealth quintile, urban residence, employment status, and ethnicity were associated with higher odds of using facility delivery. The study also found a strong and independent effect of gender, indexed by autonomy in decision-making and the possession of more modern attitudes regarding a wife’s ability to control her sexuality on the odds of using facility delivery [35].

Although many studies examined use of unskilled attendance, the phenomenon of NOP is neither well researched nor well-understood. For instance, there is no one study in Nigeria which have focused on what, where, which, or why women choose to deliver with NOP. This paper is the very first to focus exclusively on the topic and aims at using empirical data, to describe patterns, levels, and correlates of deliveries with No One Present (NOP) in Nigeria. Specifically, the study will address the following questions: what is the prevalence of delivery with NOP in Nigeria? What is the profile of women who are more likely to use this delivery approach? Where do they live and work? What is their socioeconomic status (e.g. education, residence, employment, income, and decision making autonomy)? What are the levers for NOP deliveries and what types of actions will be needed to address them. Findings from this study are expected to contribute to policy and program strategy to better address the delivery needs of women in the community.

## Methods

The analyses utilized the nationally representative 2008 Nigeria DHS data collected from 33,385 women aged 15–49 years and 15,486 men aged 15–59 years [36]. Women who had given birth in five years before the survey were questioned about maternal, newborn, child health seeking practices, including place and type of delivery assistance for any live births. In particular, these mothers were asked who assisted with the delivery of their last, next-to-last, and second from last births. The mothers were then presented with a list of both skilled and unskilled attendants to choose from. They could also pick ‘no one’ if and only if ‘no one’ was present at the delivery of each or any of the children born in the interval. These data were then used to generate a child’s file, with every live birth delivered between 2003 and 2008 constituting a record within the file. Each record was linked with the mother’s information. Each one of these records constituted the unit of analysis in data presented in this paper. To avoid potential correlation in observations among mothers who may have had more than one birth during the study period, a subsample of the 18,208 youngest children delivered within the interval were drawn for analyses.

We also addressed the difficulty of establishing temporal order of occurrence between the outcome and predictor variables, a weakness common to cross-sectional survey data. To minimize this weakness, we utilized women’s age at the time of birth of the index child. Apart from this variable, we do not have any means of determining mothers’ characteristics at the time of birth. However, by selecting a subsample of the youngest children, we improve the plausibility that mothers’ characteristics were in effect antecedent to delivery outcomes.

This study utilized variables derived from published literature on maternal health and women’s autonomy; knowledge of the context of study, and data availability (Table 3). These variables were classified into two domains: socioeconomic domain and women’s autonomy domain. Recent work by Kavita Singh and her colleagues informed the construction of women’s autonomy measures [35].

**Table 3:** Description of outcome and predictor variables.

Variables	Variable description
**Socio-demographic variables**	
Mothers age at birth	Measured in 5 year intervals: les or equal to 19, 20–24, …, 40–49
Birth order	Interval level data, measuring the sequencing of births, from the first to the last. First births are coded 1, 2^nd^ is coded 2 and in that order.
Marital status	Coded 1 if mothers reported that they were married or cohabiting at the time of the survey, otherwise, it was coded 0.
Residence (Rural/urban)	Coded 1 if urban, otherwise 0.
region of residence	Nigeria is divided into six geopolitical zones: South East, South West, South South, North Central, North East. Each was coded 1 for specific live births if the mothers reported they belong to a specific group, otherwise each of the domains was coded 0.
Mothers’ education	Coded 1if mothers reported that they had some/completed primary education, 2 if women had some/completed secondary education or higher, otherwise it was coded 0.
Wealth quintiles	Measured using ownership of household consumables; infrastructure (e.g. building type, water, electricity, toilet facilities); small equipment (e.g. telephone, TVs), and large equipment (bikes, cars, etc.). Items were coded into a relative index of household wealth, calibrated into quintiles, each representing 20% of the score, from 1(poorest) to 5(richest) quintiles. Respondents were ranked by wealth quintiles using this index.
Religion	Coded 1 if mothers reported they were Muslim and 0 if they were Christians or affiliated with other religions.
**Women’s autonomy factors:**	
Women’s social standing	Indicated by two variables: (1) current work status, and (2) property ownership. Current work status was coded 1 if mothers said they were working at the time of survey, otherwise it was coded 0. Property ownership was coded 1 if mothers were working on own/family land as opposed to rented or borrowed land, otherwise it was coded 0.
Participation in decision-making	Measured using six elements, expected to involve husband/wife participation: decisions regarding food to be cooked, visits to family/friends, purchase of large household goods, purchase of daily needs, mother’s health care, and how to spend monies husbands earned. Coded 1 if mothers said they decided all 6 issues alone or jointly with their husbands (full participation); 2 if mothers decided any one issue alone or with their husbands (some participation), otherwise the indicator was coded 0 (no participation). In the multivariate analysis, these indicators were summarized using a dummy variable coded 1 if mothers had full participation and 0 if they had some or no participation.
Inequity perpetuating gendernorms	Indicated by (1) whether mothers had co-wives (coded 1 if yes, otherwise 0); approve wife beating, and (3) approves of wife’s autonomy over her own sexuality. Approval of wife beating was coded 1 if the mother said a wife should not be beaten under any of the measured circumstances: wife goes out without telling her husband, wife neglected her children, and wife argues with her husband or burns food; otherwise it was coded 0, indicating approval of wife beating. Approval of wife’s sex autonomy was coded 1 if mothers reported that a wife was justified to refuse sex if her husband had a sexually transmitted infection (STIs), had sex with other women, or she was tired or not in the mood, otherwise it was coded 0.
Outcome variable	NOP at birth: Coded yes if the index child was delivered in 5 years before the survey (between 2003 & 2008) with NOP, otherwise NOP was coded 0.

Measures of each domain and a detailed description appear in Table 3. The socioeconomic domain factors include mother’s age, marital status, birth order, religion, place of residence (rural/urban), region of residence, women’s education, and household wealth quintile. Religion was used as an indicator of culture, which, in some studies, was observed to influence the use of unskilled birth attendance. The women’s autonomy domain is represented by three composites: women’s social standing, mother’s participation in decision-making and the prevalence of gender norms that perpetuate inequity [35], [37]. The latter was indicated by three variables: (1) whether women had co-wives, whether they are in polygynous *versus* monogamous marriages, (2) disapproval of wife’s beating and, (3) approval of wife’s autonomy over their own sexuality. Polygyny was coded as 1 if women had co-wives; otherwise, it was coded as 0. On wife beating, women were asked whether wife beating was acceptable under three circumstances: wife goes out without telling her husband, wife neglected her children, and wife argues with her husband or burns food. Mothers who said it was not acceptable to beat wife in any of these circumstances were coded as 1; all others were coded as 0. On sex autonomy, mothers were asked if a wife was justified to refuse sex with her husband if her husband had a sexually transmitted infection (STIs), had sex with other women, or when the wife was tired or not in the mood. Those who said that a wife could refuse sex under any of these circumstances were coded as 1; all others were assigned a code of 0.

Women’s social standing in the society was captured by two measures: current work status, and property ownership. Mothers who said they were working at the time of survey was coded 1, otherwise they were coded 0. On property ownership, mothers were assigned a code of 1 if they reported that they were working on own/family land, otherwise they were coded 0. Working and doing so on own or family land, as opposed to working on rented or borrowed land, was used as proxies for property ownership.

Women’s autonomy in decision making was measured with six elements, which should involve women’s participation in contexts where women had a voice. These were decisions regarding what food to cook, visits to family/friends, purchase of large household goods, purchase of daily needs, mother’s health care, and how to spend monies husbands earn. The survey questioned respondents on who had the final say on these issues. Mothers who said that they (the wife) decided on all 6 issues alone or jointly with their husbands were coded as 1 (full participation); mothers who decided any one issue alone or with their husbands were coded as 2 (some participation), otherwise they were coded as 0 (no participation). In the multivariate analysis, however, this indicator was converted into a dummy variable, coded as 1 to represent full participation in decision making, and 0 if partial or no participation in decision making. Those who reported full participation were compared with mothers who reported partial or no participation for their effects on NOP deliveries. The outcome measure, delivering with NOP, was coded 1 if mothers reported delivering their youngest children in 5 years before the survey with NOP; otherwise the variable was coded as 0.

Study data were analyzed using STATA statistical package. Estimates of types of delivery assistance were disaggregated by mother’s age at birth, birth order of children, residence, mother’s socioeconomic status and women’s autonomy factors. The significance of observed differentials was statistically validated using ordinary chi-square test and 95% confidence intervals calculated for each estimate, as proof of quality of these estimates. Multivariate logistic regression was calculated to identify critical predictors of NOP deliveries in the study population. In the analysis, we utilized the STATA’s svy command to take account of peculiar characteristics of the data, particularly the complex cluster survey design features, including sampling weights, clustering and stratification, and to ensure that these features were correctly applied in the analysis. Sampling weights were used to control for over- or under-sampling within groups.

The Nigeria DHS 2008 survey was approved by Ethics Committee of the ICF Macro at Calverton in the USA and by the National Ethics Committee in the Ministry of Health in Nigeria. To secure participants consent, an informed written consent statement was designed and attached to each individual interview. The statement was read to each selected participant at the start of the interview and their agreement was sought. All and only the participants who gave written consent participated in the survey.

### The Country, Nigeria

Nigeria occupies a land area of 923,768 Square Kilometers. It is located in West Africa, bordered in the North by Niger, Chad in the North East, Cameroon in the East, Benin in the West, and the Atlantic Ocean in the South. The country is divided into six geopolitical zones: North Central (NC), North East (NE), North West (NW), South East (SE), South South (SS), and South West. The country with its 36 states operates a Federal system of government. The 2008 Nigeria Demographic and Health Survey [36] reported that the percentage of the population living in the bottom 20% of the wealth index ranges from 5.2 in all three southern zones to 20.6% in the NC, 31.9 in NW, and 47.4% in NE. The percentage of the population in the wealthiest quintile is 32% in the South, 13.8% in NC, 7.6 in NW, and 3.2% in NE. The percentage of women currently working ranges from 64% in the three southern States to 62.8% in NC, 57.1% in NE, to 46.0% in NW. Twenty-two percent of the population in the North versus 43% in the South is urban. Nationally, total fertility rate (TFR) is 5.7 children. In the northern states combined, the TFR is 7. It is 5.2 in the southern zones. Nationally, the infant mortality rate (IMR) is 87 per 1000 live births; 79 in the southern zones combined, 77 in NC, 109 in NE, and 91 in the NW respectively, per 1000 live births. More than half of the IMR is contributed by neonatal deaths, suggesting that maternal causes contribute disproportionately to the observed high IMR in the country [1]. In the Northern parts of the country, the MMR is well over the national average of 630/100,000 live births. For example, Zamfara State has a rate of 1049/100,000 live births [8] and that for Sokoto State is 1500/100,000, which is among the highest rates reported in the country [7].

## Results

**Figure 1:**
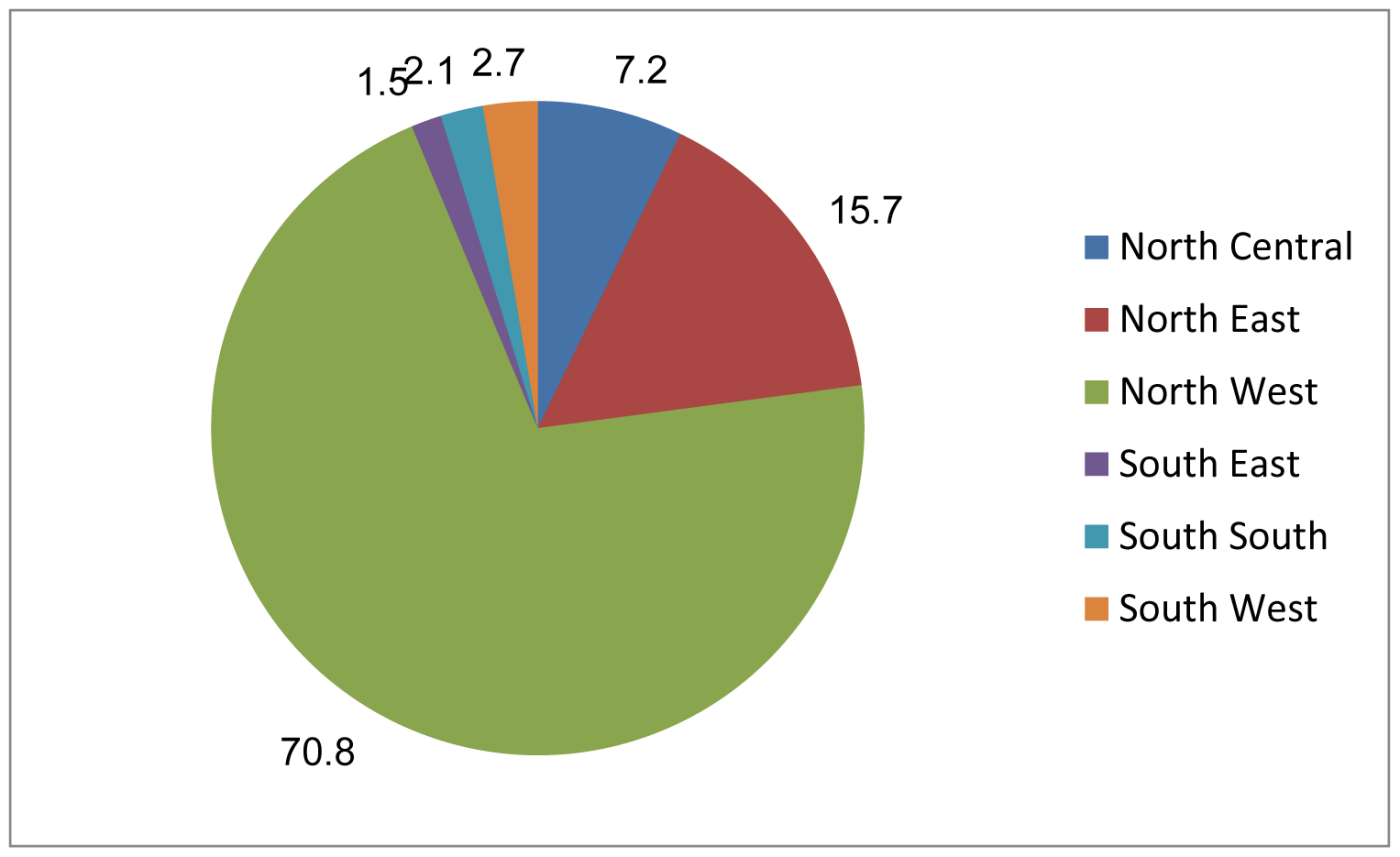
Political Zone: North Central, North East, North West, South East, South South, and South West.

Figure 1 presents the distribution of all live births delivered with no one present (NOP) in the five years preceding the NDHS 2008 survey. As shown, most of these births were located in the Northern part of Nigeria. Indeed 94% of the NOP births were located in the country’s three northern political zones. The North West alone had roughly 70% of all the cases while the three southern zones had about 6% of the total cases of children delivered with NOP in 5 years preceding the survey.

The unweighted number of livebirths included in the analysis are presented in Column 2 of Table 4. The weighted percentages along with the 95% Confidence Intervals are presented for each of the measured dimensions (Column 3). As shown in Column 3 of Table 4, the mothers were normally distributed by age, roughly 70% were within 20–34 age group. Most of the mothers were currently married, predominantly rural and poor; about 64% were within the bottom 3 quintiles of the wealth index. About half of the mothers had no formal education. Among those with formal education, 58.3% of them attained primary school level. Most of the births were higher order births, that is second, third or higher. More than one third of all the births were in the 5^th^ or later birth orders. Mothers were almost equally split between being Christian and being Muslim. Less than 3% of the mothers practice other religions. The 95% CI substantiate the validity and credibility of these estimates.

**Table 4:** Distribution of all live births and births delivered with NOP in 5 years preceding the survey by mother’s socioeconomic characteristics, Nigeria DHS 2008.

Characteristic	Unweighted live births, 5 yrs preceding survey	Weighted %	95% CI		Weighted % delivered with No One Present	Chi2
			From	To		
**Age**						
15–19	1,818	9.7	9.73	9.73	18.8	
20–24	4,000	21.8	21.78	21.79	16.3	
25–29	4,821	26.8	26.82	26.83	17·6	
30–34	3,414	19.6	19.63	19.64	18.5	
35–39	2,479	13.8	13.87	13.87	21.0	
40+	1,496	8.2	8.15	8.15	29.0	109.24***
**Marital status**						
Single/widowed/divorced	1,003	5.4	5.44	5.44	7.6	
Currently married	17,025	94.6	94.55	94.55	19.7	89.23***
**Birth Order**						
1	3,661	17.3	17.31	17.31	8.8	
2	2,943	16.6	16.59	16.59	14.2	
3	2,719	15.3	15.34	15.34	16.3	
4	2,372	13.3	13.33	13.33	17.6	
5+	6,933	37.4	37.43	37.43	27.5	439.83***
**Residence**						
Rural	13,202	70·0	69.77	69.77	23·5	
Urban	4,825	30.0	30.22	30.22	8.6	409.86***
**Religion**						
Christian	7,604	43.8	43.77	43.77	3·6	
Islam	9,955	55·0	54.95	54.95	31·2	
Other(incl Traditionalists)	469	2.3	2.28	2.28	28·1	180·00***
**Region**						
North Central	3350	14.3	14.32	14.32	10·9	
North East	3972	15.6	15.60	15.60	19.1	
North West	4888	30.5	30.46	30.46	43.5	
Southern Zones	5818	39.6	39.61	39.61	3·0	290.00***
**Education**						
No education	8870	45·5	45·46	45·46	34·3	
Primary	4062	22·2	22·75	22·75	10·7	
Secondary+	5096	31·8	31·79	31·79	3·2	190·00***
**Wealth Index**						
Poorest	3256	23·1	23·10	23·10	35.0	
Poorer	3180	22·2	22·21	22·21	27·6	
Poor	3007	19·0	19·00	19·00	15·5	
Richer	2836	18.2	18.17	18.17	7·5	
Richest	2406	17.5	17.53	17.53	2.9	140·00***
**Total**	**18,028**	**100**			**19·0**	

*** P<0.01.

**Figure 2:**
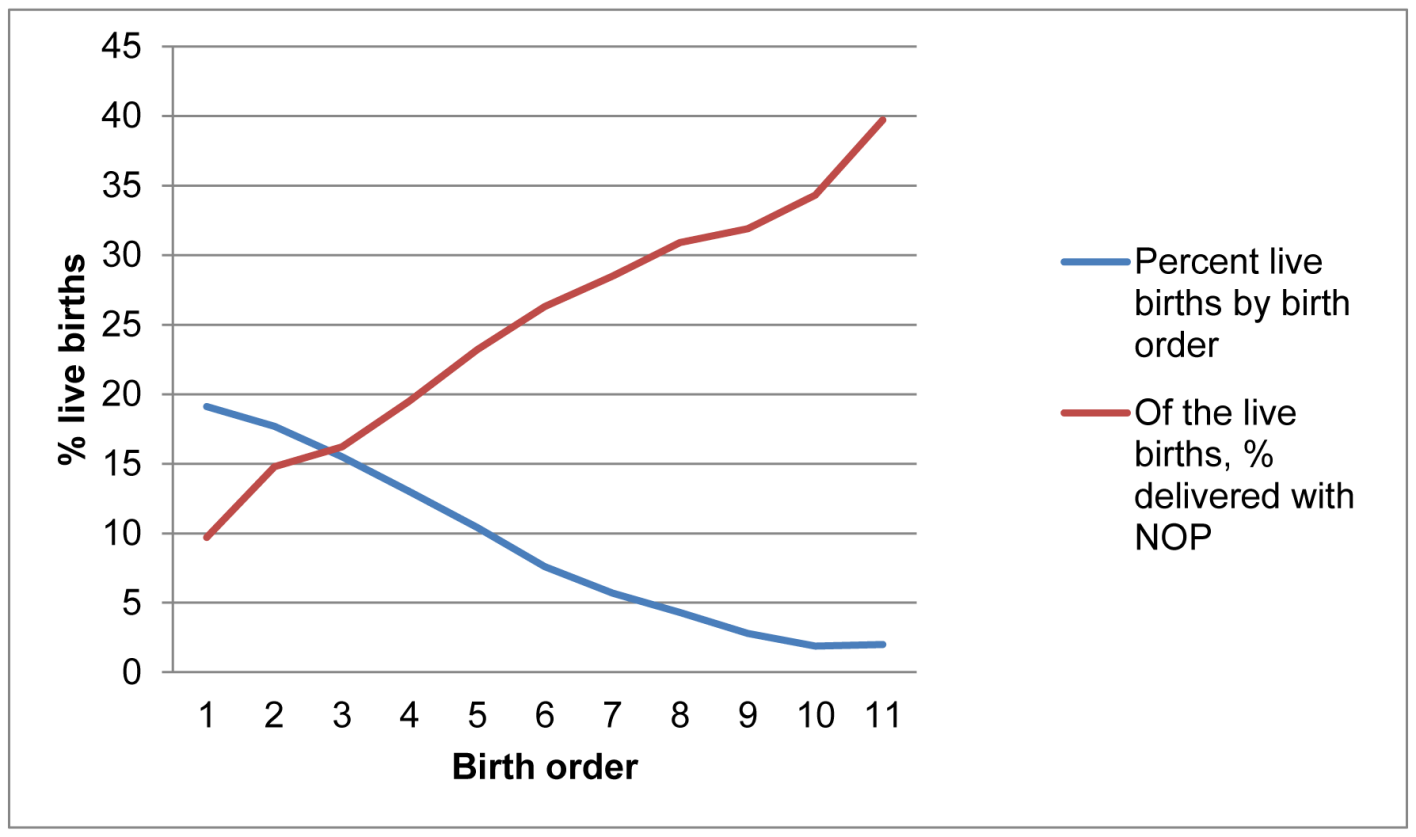
Percent live births; of the live births, % delivered with NOP.

The weighted distribution of live births by delivery assistance received is presented in Column 4 of Table 4. As shown, the proportion of live births delivered with NOP in 5 years preceding the survey is higher for older, married, rural, poor, Muslim mothers and among mothers who live in the northern part of the country. The proportion of NOP births among mothers with no education was triple that of mothers with primary education and was 10 times greater than NOP births among women with secondary education or higher. In Figure 2, we examined the association between children’s birth order and NOP delivery. Birth order is defined as the sequencing of births; lower order births are births which occur early in the reproductive career, e.g. births which occurred in the 1^st^, 2^nd^, or 3^rd^ positions in the family. Having these children is of high priority for young women who must prove their womanhood and establish their position in their marriages. Higher order births are births in the 4^th^, 5^th^, 6^th^ or higher positions. Within the value of children theory, posited by John Caldwell, these births do not carry as much weight as lower order ones because, by the time these are born, mothers are no longer under pressure to prove their fertility or self-worth. The use of this concept began with African demography and it captures elements of a cultural regime in which children and child bearing is a defining feature in the valuation of women within and outside of the household [38].

Consistent with the theory, the percentage of children born with NOP rose sharply with an increasing birth order. In other words, children who were 4^th^,5^th^, or 6^th^ order births in their families,were more likely to be born with NOP than those who were 1^st^, 2^nd^ or 3^rd^ order births.

With respect to equity, about a third of the live births in the poorest wealth quintiles were born with NOP. The results show that the probability of being born with NOP appeared to fall with marginal increments in wealth quintile. To elaborate this association, we collapsed wealth index into two categories; mothers in one of three lower quintiles of the original index (poor, poorer, poorest) were coded as 1, and those in upper quintiles, richer/richest categories, were coded as 2. The result (not presented) showed that children from poorer households were 5 times more likely delivered with NOP than those from richer households. The inverse relationship between income and livebirths with NOP may partly explain why NOP deliveries in rural areas were nearly triple those in the urban areas; urban residents tended to place higher in the economic quintiles. We conducted a chi-square test to confirm the significance of these associations and the findings, reported in Table 4, Column 7, showed that all observed associations were statistically significant at the ·001 alpha level.

Next, we assessed the effects of women’s autonomy on the likelihood of NOP in the five years preceding the survey (Table 5). Several studies have shown that women’s autonomy indicators have signifcant independent effects on reproductive care choices, including the place and type of delivery attendance. As shown in this study, when women’s autonomy was high, children were less likely to be born with NOP than when their autonomy was low. In all, the proportion of livebirths delivered with NOP was small if mothers participated in decision-making in the household, felt it was never okay to beat wife, or felt that women have the liberty to refuse sex if they were tired, not in the mood, or their husbands had STIs or were engaged in sex with other women.

**Table 5:** Distribution of all live births and births delivered with no one present in 5 years preceding the survey by mother’s autonomy characteristics, Nigeria DHS 2008.

Characteristic	Unweighted No of live births, 5 yrs preceding survey	Weighted %	95% CI		Weighted % delivered with No One Present	Chi2
			From	To		
**Current work status**						
Current working	11568	65·4	65·46	65·46	15·7	
Not working	6,460	34·6	34·54	34·54	25·4	192.42***
**Property ownership** #						
Work on personal or family land	2,728	12.9	12.89	12.89	9·2	
Work on rented or borrowed land	8,840	87.1	87.11	87.11	17.2	73.69***
**Mothers have co-wives [yes, no]**						
Yes	6804	36.22	36.23	36.23	25·0	
No	11,224	63.8	63.77	63.77	15·6	140.01***
**Decision making autonomy**						
Women take all 6 decisions alone or jointly with their husbands	3,484	19.0	19.02	19.02	11·6	
Wife participates in any one of 6 decision elements	6,961	39.6	39.56	39.56	13·8	
Wife participates in No decision	7,583	41·4	41·42	41·42	27.4	334.60***
**Attitudes towards wife beating**						
Never ok to beat wife for any reason	8,574	49·1	49·11	49·11	14·0	
Ok to beat wife for at least one reason	9,454	50.9	50.89	50.89	23·9	249.83***
**Wife’s sex autonomy**						
Wife has right to refuse sex for any of the three reasons	8,211	46.6	46.55	46.55	14·8	
Wife has no right to refuse sex forany reason	9,817	53.4	53.45	53.45	22.7	132.90***
**Total**	**18,028**	**100**			**19.0**	

# based on number of mothers who reported that they were working.

*** P<0.01.

Findings on other indicators of women’s autonomy, including whether the mothers were currently working and, if yes, whether they were working on own or family land, are also presented in Table 5. As expected, we found mothers who were currently working and doing so on own or family land were less likely to give birth with NOP than their counterparts who were not working, do not have land, or were in polygynous relationships. A chi squared test found these associations to be statistically significant at the ·001 alpha level (Table 5, col 7).

In Table 6, we re-examined these relationships using multivariate logistic regression analyses. Three models were assessed. Model 1 included all the socioeconmic variables. Model 2 consisted of all the women’s autonomy factors. Both of these domain indicators (socioeconomic and women’s autonomy factors) were included in Model 3 (Table 6, Col 4). The result showed that four factors: mother’s age at birth, birth order of children, religion, and region of residence substantially increased the likelihood of being born with NOP. However, mothers education, residence, and wealth quintile, protected against NOP deliveries. Being married or single did not appear to determine whether a child was likely or not to be born with NOP. However, mothers with primary or secondary education, respectively, were 22% and 54% less likely to give birth with NOP than mothers with no formal education. Similary children of wealthier mothers were significantly less likely to be born with NOP than those from poorer households. Indeed, with a marginal increase in the wealth quintiles, the odds of being born with NOP decreased substantially. For instance, live births delivered to mothers in the 3^rd^, 4^th^ and 5^th^ wealth quintiles, respectively, were 46%, 66% and 77% less likely to be born with NOP.

**Table 6:** Multivariate analysis of deliveries with no one present (NOP) by socioeconomic and mother’s autonomy factors, live births in 5 years preceding the Survey, Nigeria DHS 2008.

Characteristic	Model 1	Model 2	Model 3
Socioeconomic Characteristics			
**Age**			
15–24	Reference		Reference
25–29	1·31***		1·31***
30–34	1·25***		1·23***
35–39	1·24***		1·23***
40+	1·36***		1·36***
Marital status			
Currently Married/Cohabiting	Reference		Reference
Single (never married/not cohabiting/divorced/widowed)	.99***		1.00***
**Birth order**	1.10***		1.09***
**Religion**			
Tranditionalist/Other	Reference		Ref
Islam	2·10***		1·77***
**Residence**			
Rural	Reference		Reference
Urban	·97***		·97***
**Education**			
No education	Reference		Reference
Primary	·68***		·68***
Secondary+	·46***		·47***
**Wealth Index**			
Poorest	Reference		Reference
Poorer	·74***		·72***
Poor	·54***		·52***
Richer	·34***		·33***
Richest	·23***		·22***
**Region**			
Southern Zones	Reference		Reference
North Central	1·72***		1·81***
North East	1·68***		1·75***
North West	6·04***		5.92***
**Women’s Autonomy Indicators**			
**Women’s standing in the society**			
Not working		Reference	Reference
Currently working		·65***	·97***
Work on rented or borrowed land		Reference	Reference
Work on personal or family land		·45***	·57***
**Mother’s had co-wives [yes, no]**			
Yes		Reference	Reference
No		·61***	·87***
**Attitudes towards wife beating**			
Ok to beat wife for at least one reasons		Reference	Reference
Never ok to beat wife for any reason		·54***	·78***
**Wife’s sex autonomy**			
Wife has no right to refuse sex		Reference	Reference
Wife has right to refuse sex		·66***	·83***
**Decision making autonomy**			
Participate in some or no decisions		Reference	Reference
Participate in all decisions		·66***	.98***
Participate in all decisions X birth order			1.04***

*** P<0.01.

The regression analysis results inclusive of the association of women’s autonomy measures with NOP delivery is presented in Table 6, Model 2. As shown, all six indicators had negative effects, which means that they protected against giving birth with NOP. Mothers who worked, did so on own/family land, were involved in household decisions, who considered any form of wife beating as unacceptable, considered a wife’s right over her own sexuality as justifiable, and were in monogamous marriages, had lower odds of delivering with NOP than their counterparts who had limited social standing, limited decision-making autonomy, and/or possess higher doses of norms that amplified inequities.

In column 4 of Table 6, we presented the full model. In this model, all socioeconic indicators remained statistically significant, almost at the same level and direction as observed in Model 1. Mother’s age at birth, birth order of children, religion, and region of residence continued to increase the odds of being born with NOP. The effect of mother’s education, urban residence, and wealth quintile were negative, indicating that these factors protected against being born with NOP. So also were the efffects of women’s autonomy measures. Children were less likely to be born with NOP if they were born to mothers who work, work on own land, approve of women’s control over their own sexuality, disapprove of wife beating, and participates in decision making in the household. The magnitude of the effects of women’s autonomy variables, however, declined in this model though they were still statistically significant. We also found statistically significant interaction between mother’s decision-making autonomy and birth order. Specifically, the observed negative association between the odds of NOP deliveries and autonomy in decision making varied by birth order. This effect suggests that as family size grows, women who participates in decision-making and who ordinarily would have been expected to opt for less risky behavior, tended to have their babies at home on their own with no one present.

## Discussion

Maternal mortality is still unacceptably high in Nigeria. Although some declines have been observed, the current ratio is still double the regional rate for developing countries. Use of skilled attendance at birth has been shown to be a key to maternal mortality decline, but coverage of births by skilled attendance is one of the most inequitable health interventions in Nigeria. Not only are women using unskilled attendants, an unacceptable 1 in 5 births are delivered with NOP. Even far more striking is the fact that there is very little known about the socioeconomic profiles of these mothers and their children. Such information will be crucial to designing programs that tailor interventions to eliminate this practice. A key objective of this paper was to produce this information.

The study data were obtained from Nigeria Health and Demographic Survey (NDHS) conducted in 2008. NDHS is a representative survey of women of reproductive age in Nigeria. The study found that all socioeconomic and women’s autonomy factors examined were statistically significant. Of the socioeconomic factors, mother’s age, birth order, region of residence, and being Muslim were instrumental in increasing the odds of children being delivered with NOP. Other factors such as urban residence, women’s education, and household wealth index, reduced the odds of children being delivered with NOP. Similarly, the indicators of women’s autonomy, including women’s work status, land ownership, decision making autonomy, and measures of gender norms that perpetuate inequity were statistically significant in reducing the odds that children will be delivered with NOP.

These findings suggest three potential explanations for the prevalence of NOP in the country: (1) an economic security argument, (2) a women’s autonomy argument, and (3) a cultural argument. With respect to the first argument, the findings indicate that economic security is a key factor in whether or not children will be born with NOP. We found that economic factors such as wealth quintiles, mother’s education, women’s employment and property ownership offerred significant protection against NOP deliveries. Given the relatively high cost of formal health services in Nigeria, these findings suggest that mothers who were economically insecure were less likely to take advantage of available services.

On the second argument, we found that women’s viability, visibility, and worth, branded as women’s autonomy, indicated by their ability to participate in decision making, own property, able to overcome inequity pepetuating tendencies, and maintain control over their own sexuality, protected against delivery with NOP. The magnitude of these effects declined once the socioeconomic factors were included in the model equation, but nonetheless remained statistically significant. In effect, although women’s autonomy and social standing are critical to decisions regarding the choice of delivery attendance, these effects were attenuated by the degree of economic prosperity. Therefore, in resource poor contexts, women who are less properous are less able to apply the necessary gravitas required to influence decisions and legitimize their autonomy. In addition, they lack the means to execute correct actions that they know will lead to better outcomes. However, more prosperous women more likely possess the requisite capabilities to secure spousal respect, make their own voices heard, and to act on them. Economic power provides an important lever for women to use their autonomy to effect behavior change. A woman who has economic power to finance essential actions, also has the autonomy to make decisions. Without economic power, women, even with decision making power, may resort to less optimal delivery choices such as have children at home, on their own, or with no one present rather than with skilled attendance. This is illustrated in this study by the significant interaction effect of birth order and mothers participation in decision making on NOP deliveries. In other words, as birth order increased, children were more likely born with NOP than with skilled care even among women with decision autonomy. It is common knowledge that, in resource-poor settings, mothers with large numbers of children are likely more economically challenged than those with fewer children. So, there is a clear value in improving women’s economic base by investing in education, jobs, faciliating property ownership and increasing access to factors of production. When women have access to these resources, their social and economic standing are enhanced and their voices command more respect at the family and at the sociental levels. They are also able to use economic purchasing power to demand optimum delivery services.

The third explanation centers around the influence of culture and traditions as key players in women’s choices of delivery care. Although our data do not offer a way to directly test this arguement, the observed effects of contextual factors such as region of residence, religion, birth order, and age of mothers on NOP deliveries are illustrative. In this study all the measured contexual factors increase the odds of deliverying with NOP. Indeed, this domain contributed the most to the explanation of why women deliver with NOP. The likelihood that a live birth was delivered with NOP increased by 9% with every additional delivery after the first birth. Being Muslim almost doubled the odds that a specific child will be born with NOP. Region of residence is by far the most influential of all the factors studied. Mothers who were resident in the North West were six times more likely to deliver with NOP than their counterparts in the South; they were 4 times more likely than women from other northern regions. Although, not a contexual variable per se, age increased exposure and dose level of specific interventions and norms. In our specific instance, older women will be more likely to have a greater dose of the injurious beliefs embedded in their cultural context and are more liable to have these affect their behavior than younger women. In the absence of qualitative information on these contexual factors, we will not know which of these factors is operative or how they interact to shape women’s choices. We can surmise that each of these factors captures beliefs systems that are supportive of more traditional and more conservative, informal, practices that are somewhat adversarial to women’s health. For instance birth order is a proxy for value of children and how children are valued is influenced by culture. Per this study, higher order births are more likely to be delivered with NOP than lower order births. This practice may reflect a declining value of children as family size increases. Or it could also be that women who have had babies already now consider themselves to be veterans who need no help. In addition, the North West, where 70% of NOP deliveries come from, also happen to be the region of the most conservative form of Islam and traditions which define tolerance for pain as a measure of strength, and risk taking as laudable acts of bravery. It is not uncommon, therefore, that in this setting, women will opt for home delivery with NOP rather than use skilled attendance. This would most likely be considered heroic and brave. Therefore, efforts to improve delivery practices will have the greatest result potential in this region by identifying catalytic elements in the culture that are injurious to women’s health and developing behavior change modification messages to address these elements.

In improving the economic situation of women, we see the short-term and longer-term considerations. The longer-term include investments in education, promoting entrepreneurship and societal eradication of poverty. The shorter-term interventions linked to speedier improvements in maternal health outcomes, and linked to helping pregnant mothers to finance costs associated with health care, will benefit from the use of conditional cash transfers (CCT) to increase mothers’ effective demand for safer maternal health services. CCT has been piloted in many countries in Latin America (e.g. Mexico; Nicaragua; Honduras), South America (e.g. Brazil; Colombia), and 18 countries in sub-Saharan Africa, including Eritrea, Mozambique, and Senegal [39], [40], [41]. The intervention has been proven to increase demand and use of maternal and delivery services in these contexts. Recently in Nigeria, unpublished reports from the Nigeria Ministry of Health of a pilot study in two wards in the Abuja Federal Capital Territory (FCT) area suggest that CCT played a pivotal role in relaxing the barrier of transportation costs and the costs of services to mothers, increasing the utilization of services in the two wards (see Pate M, Oshin A, 2012. SURE-P Maternal and Child Health Project: Conditional Cash Transfer Programme: Evidence for Action in two Wards in Abuja, FCT). Use of CCT will, however, have to be applied with caution so that it does not reverse the gains in fertility decline by motivating mothers who ordinarily would have ceased child-bearing to continue to have children.

A key weakness in this study is its exclusive focus on demand side, population level, factors in explaining delivery care practices. The main reason for this focus is the absence of access related measures in the NDHS to elaborate the context of service delivery, that is, the readiness of health facilities to provide services and how this contributes to the choice of delivery assistance. This is a key area worthy of further investigation. Finally, the finding that the North West has 70% of the national burden of delivery with NOP is instructive. Presently, there is, as of now no analysis that has focused exclusively on this region in order to uncover the reasons for such a high prevalence of NOP deliveries. Understanding the local context is crucial for strengthening delivery care and services in the country. This should be a key focus for further research.

In conclusion, there is an urgent need to eradicate the practice of NOP deliveries if Nigeria is to accelerate and sustain improvements in maternal health per the millennium development goal (MDG 5) [42]. In addition to employing short-term financial instruments such as conditional cash transfer, systematic investments in improving women’s autonomy and economic security through education and social entrepreneurship for mothers should be prioritized. Efforts also need to focus on identifying potential cultural/religious resources that can be used to promote positive health seeking behavior. The supply of quality health services will greatly aid the uptake of these services.
